# *Xylella fastidiosa*: A reemerging plant pathogen that threatens crops globally

**DOI:** 10.1371/journal.ppat.1009813

**Published:** 2021-09-09

**Authors:** Claudia Castro, Biagio DiSalvo, M. Caroline Roper

**Affiliations:** Department of Microbiology and Plant Pathology, University of California, Riverside, California, United States of America; THE SAINSBURY LABORATORY, UNITED KINGDOM

*Xylella fastidiosa* is a fastidious, gram-negative bacterium in the family Xanthomonadaceae and is a major threat to agricultural crops and ecological and ornamental landscapes in the world. This bacterium is quite remarkable in regard to its very broad host range that includes over 600 plant species belonging to 63 diverse plant families. It is specifically limited to the xylem tissue of its plant hosts [1]. In some of these hosts, it causes severe and devastating disease. However, in the vast majority of its hosts, it is considered a benign commensal.

*X*. *fastidiosa* is endemic to the Americas. Historically, Europe was considered to be free of *X*. *fastidiosa*, but the bacterium was recently detected in Italy. In 2013, olive trees in the Apulia region of Southern Italy began exhibiting leaf scorch symptoms that were later confirmed to be caused by *X*. *fastidiosa*. Since then, thousands of olive trees have died, and *X*. *fastidiosa* has been detected in various plants species in France, Spain, and Portugal [1–3]. *X*. *fastidiosa* has been responsible for significant economic losses in regions like the United States, Italy, and Brazil. For example, *X*. *fastidiosa* subsp. *fastidiosa*, the causal agent of Pierce’s disease (PD) of grapevine, leads to crop losses of approximately US$104 million and costs growers approximately US$50 million in preventative strategies each year for the California viticulture industry [4,5]. In the Apulia region, *X*. *fastidiosa* subsp. *pauca* infection in olive orchards is projected to cost Italy up to €5.2 billion over the next 50 years if trees are not replaced [6]. Current management strategies to minimize *X*. *fastidiosa* spread in the field include removal of infected plants, severe pruning, and control of insect vectors with insecticides. The development of resistant plant lines is also an active area of research, and, recently, 5 new PD-resistant grape varieties were commercially released to the grape industry [7].

The *X*. *fastidiosa* species is subdivided into multiple subspecies that include subsp. *fastidiosa*, *multiplex*, and *pauca* [8]. The subspecies designations are loosely associated with host range, but some strains can infect multiple hosts. In general, disease symptoms associated with these *X*. *fastidiosa* strains are most commonly characterized by marginal leaf necrosis or leaf scorching like those observed in grapevines infected with *X*. *fastidiosa* subsp. *fastidiosa*. However, symptoms caused by *X*. *fastidiosa* subsp. *pauca* can be characterized by foliar wilt and interveinal chlorosis, and symptoms caused by *X*. *fastidiosa* subsp. *multiplex* in some hosts can exhibit dense canopies and reduced fruit size [1]. *X*. *fastidiosa* has no free-living component of its lifestyle and has only been found associated with its plant and insect hosts.

## *Xylella fastidiosa* has a unique association with its xylem sap–feeding insect vectors

**Fig 1:**
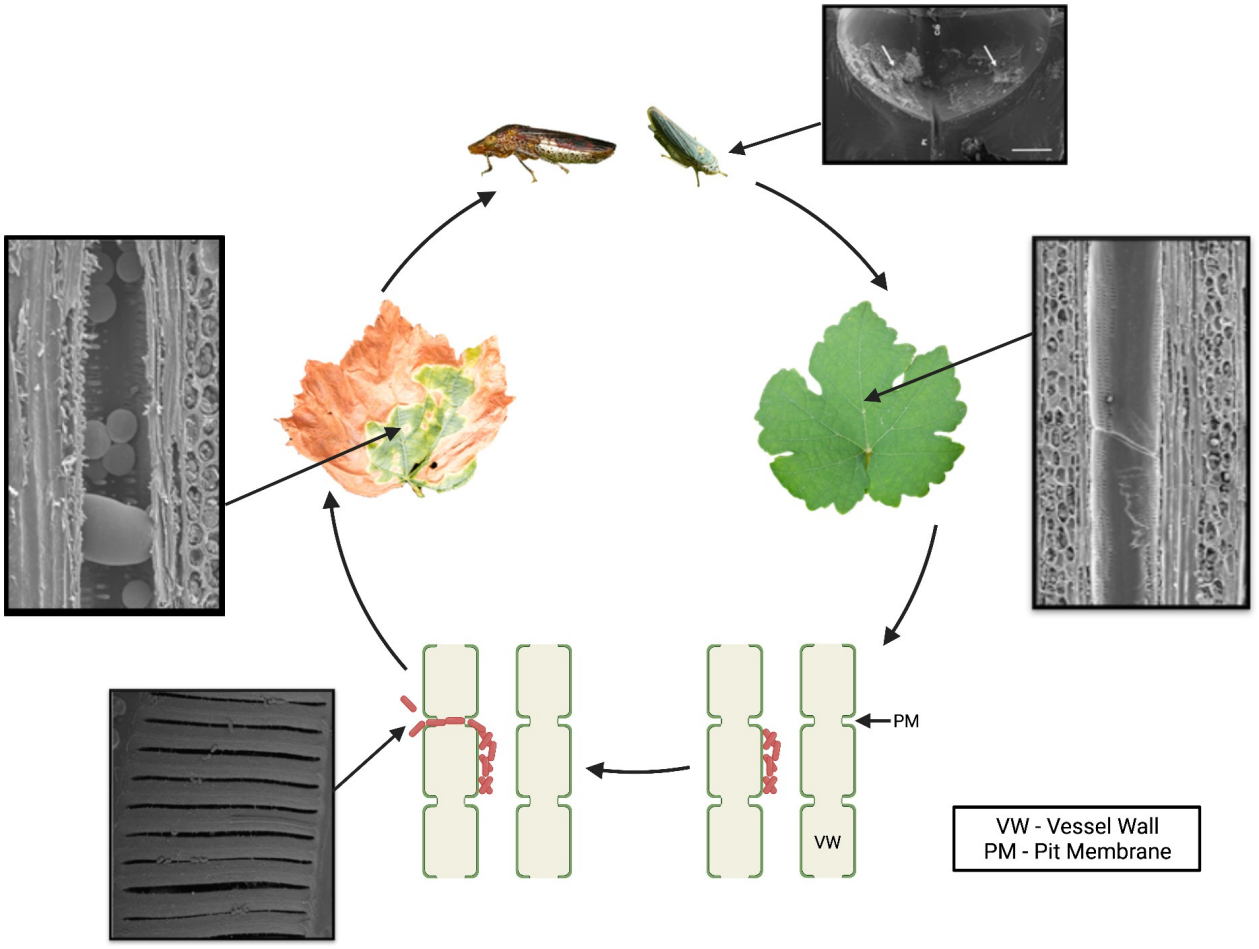
PD of grapevine cycle. *Xylella fastidiosa* is acquired by its xylem-feeding insect vectors, such as the GWSS and the BGSS, during the feeding process. Once acquired, it colonizes the insect’s foregut and forms robust biofilms (indicated by white arrows). *X*. *fastidiosa* is transmitted to a new host plant when the insect vector feeds on a new plant and deposits *X*. *fastidiosa* cells directly into the plant xylem. *X*. *fastidiosa* achieves systemic colonization of the xylem by enzymatic degradation of the xylem pit membranes that connect adjacent xylem vessels. *X*. *fastidiosa* colonization induces prolific production of balloon-shaped defense-related protrusions called tyloses in the xylem. Systemic colonization and vessel occlusion by bacterial biofilms and excess tylose production lead to PD symptom development. *Photo credit for the BGSS*: *Rodrigo Krugner*. *Photo credit for the xylem longitudinal sections*: *Qiang Sun*. *Pit membrane photo reprinted from Ingel et al*., *2019*, *Molecular Plant-Microbe Interactions Vol*. *32*, *No*. *10*: *1402*–*1414*. *Insect foregut image reprinted from Rapicavoli et al*., *2015*, *Applied and Environmental Microbiology Vol 81*, *No*. *23*: *8145*–*8154*. Created with BioRender.com. BGSS, blue-green sharpshooter; GWSS, glassy-winged sharpshooter; PD, Pierce disease; PM, pit membrane; VW, vessel wall.

*X*. *fastidiosa* is obligately vectored by xylem-feeding hemipteran insects primarily belonging to the sharpshooter leafhopper (Cicadellidae) and spittlebug (Cercopidae) families (Fig 1) [9–11]. These insects are polyphagous (i.e., they feed on many plant species) and are present in warm regions across the globe [11]. *X*. *fastidiosa* is acquired when the insect feeds on the xylem sap of an infected plant. The bacteria colonize and multiply in the insect foregut (mouthparts) in a persistent, but noncirculative manner [10,12]. This type of pathogen–vector relationship is unique among insect-vectored plant pathogens because the bacterial cells propagate within the insect mouthparts but do not circulate throughout the body of the insect, whereas most propagative pathogens circulate within the insect. When sharpshooters feed on the xylem of infected vines, *X*. *fastidiosa* attaches to and colonizes the insect foregut where it forms adhesive biofilms (Fig 1). *X*. *fastidiosa* experiences extreme shear stress during the xylem sap ingestion and egestion processes that occur during insect feeding. During transmission into a healthy vine, bacterial cells dislodge from the insect foregut, presumably as a result of the high shear stress created during feeding, and are deposited directly into the xylem of healthy vines [13]. There is no apparent specificity between a particular *X*. *fastidiosa* subspecies and insect vector species. In fact, individual glassy-winged sharpshooter (GWSS) (*Homalodisca vitripennis*) can acquire more than 1 *X*. *fastidiosa* subspecies in its foregut and can potentially transmit these strains to a variety of plants where the bacterium can behave as pathogen or a commensal endophyte [2,14].

In the context of PD of grapevine caused by *X*. *fastidiosa* subsp. *fastidiosa*, the pathosystem with the broadest literature base, the 2 xylem-feeding insects transmit *X*. *fastidiosa* that have received the most research focus are the blue-green sharpshooter (BGSS) (*Graphocephala atropunctata*) and the GWSS. The BGSS is native to riparian areas in California and feeds on new plant growth that emerges in the spring [9,10]. The GWSS is invasive to California and was introduced into Southern California approximately in 1989 [15]. The introduction of this invasive pest drastically changed the epidemiology of PD in the southern part of California because GWSS can feed on both green and dormant woody tissues, enabling transmission even in winter. In addition, GWSS can fly longer distances than native sharpshooter species, which could explain how PD incidence was elevated to epidemic proportions in Southern California. Subsequently, there has been a concerted effort among growers and the California Department of Food and Agriculture to control vector populations and prevent the spread of GWSS. The predominant vector linked to olive quick decline syndrome in Italy is the meadow spittlebug, *Philaenus spumarius* [16].

## *Xylella fastidiosa* colonizes host compartments that are primarily nonliving

As far as presently known, *X*. *fastidiosa* interacts primarily with nonliving tissues in both its insect and plant hosts. These include the cuticular surface of the insect foregut and the plant xylem, which is nonliving at maturity (Fig 1). The xylem consists of a network of vessels that are connected by pit membranes. These are thin, porous structures composed of primary plant cell wall, which allow for the passage of water but prevent the movement of pathogens and air embolisms. *X*. *fastidiosa* produces plant cell wall–degrading enzymes, a polygalacturonase and several endoglucanases, which act in concert to degrade pit membranes, allowing *X*. *fastidiosa* to breach this barrier and move from vessel to vessel to achieve systemic colonization [17–19]. *X*. *fastidiosa* is also a prolific producer of outer membrane vesicles that also modulate xylem colonization [20]. Interestingly, *X*. *fastidiosa* does not possess a type III secretion system (T3SS) typical of other pathogenic bacteria that enables them to inject cognate type III effectors into living host cells, likely because the bacterium interacts primarily with nonliving cells. Instead of relying on T3SS effectors to bypass host immunity, *X*. *fastidiosa* delays early plant recognition in grapevines by camouflaging itself with a rhamnose-rich O antigen, the most external portion of its lipopolysaccharide layer as one mechanism that allows it to skirt initial triggering of the grape immune system to establish itself in the plant [21]. It is not known which living plant tissues are primarily responsible for initiating and propagating a response to *X*. *fastidiosa*, but it is likely the living xylem parenchyma cells adjacent to the xylem vessels.

One of the remarkable internal symptom phenotypes of infected grapevines is the prolific production of tyloses in response to *X*. *fastidiosa* colonization of the xylem (Fig 1). Tyloses are outgrowths of the living xylem parenchyma cells that protrude into the xylem and are part of the plant defense response. Their role, in part, is to slow or prevent pathogen movement within the xylem. However, overproduction of tyloses can cause a reduction in hydraulic conductivity within the xylem that is detrimental to the plant [22,23]. In PD-infected vines, tyloses become the dominant form of xylem occlusion during the early stages of disease, and, as a consequence, infected vines have a significant loss in hydraulic conductivity. Tyloses exacerbate PD symptoms, and it is thought that this uncontrolled production of tyloses is what ultimately leads to the demise of the plant [24].

Another notable feature of *X*. *fastidiosa’s* behavior in planta is the manner in which it regulates its own biofilm formation as it colonizes the xylem. In general, entering into and maintaining robust biofilms are linked to promoting virulence for many bacterial pathogens [25]. On the contrary, mutant strains of *X*. *fastidiosa* that are impaired in biofilm formation and effectively locked in a planktonic phase have a hypervirulent phenotype in grapevines [26–29]. Thus, it is speculated that *X*. *fastidiosa* enters the surface adhesive biofilm state as a means to attenuate its own virulence by controlling its movement in planta by adhering to the xylem wall. This self-limiting behavior during parasitism in symptomatic/susceptible hosts may be a remnant from its lifestyle as a commensal in nonsymptomatic hosts, where tightly regulating and limiting rapid movement in the plant would promote a commensal interaction rather than a parasitic interaction.

## *Xylella fastidiosa* acts as both a commensal and a pathogen depending on its host environment

The bulk of the research on *X*. *fastidiosa* is biased toward isolates that are pathogenic in economically important hosts. The mechanism by which *X*. *fastidiosa* causes disease only in certain hosts, but not others, has not been fully elucidated, and its interactions with commensal hosts is largely understudied. However, it is speculated that compatibility between xylem pit membrane carbohydrate composition and *X*. *fastidiosa*–secreted cell wall–degrading enzymes mediate disease onset and progression [19,30]. In addition, the O antigen is a critical component in evading initial immune recognition in the susceptible grapevine immune system, and it is tempting to speculate that O antigen composition dictates the type of symbiotic association with the plant commensalism versus parasitism [21]. Understanding the mechanisms that underlie how different *Xylella*–plant host interactions skew toward parasitism or commensalism is an area of research that is ripe for exploration.
